# Preservation Methods in Isolates of *Sporothrix* Characterized by Polyphasic Approach

**DOI:** 10.3390/jof9010034

**Published:** 2022-12-25

**Authors:** Vanessa Brito de Souza Rabello, Danielly Corrêa-Moreira, Cledir Santos, Tatiana Casto Abreu Pinto, Anna Carolina Procopio-Azevedo, Jéssica Boechat, Rowena Alves Coelho, Rodrigo Almeida-Paes, Gisela Costa, Nelson Lima, Rosely Maria Zancopé-Oliveira, Manoel Marques Evangelista Oliveira

**Affiliations:** 1 Laboratory of Mycology, Evandro Chagas National Institute of Infectious Diseases, Fiocruz. Av. Brasil, 4365, Manguinhos, Rio de Janeiro 21040-360, Brazil; 2 Postdoctoral in Clinical Research in Infectious Diseases, Evandro Chagas National Institute of Infectious Diseases, FIOCRUZ, Rio de Janeiro 21040-360, Brazil; 3 Taxonomy, Biochemistry and Bioprospecting of Fungi, Oswaldo Cruz Institute, FIOCRUZ, Rio de Janeiro 21040-360, Brazil; 4 Department of Chemistry Science and Natural Resources, Universidad de La Frontera, Temuco 4811-230, Chile; 5 Instituto de Microbiologia Paulo de Góes, Universidade Federal do Rio de Janeiro, Rio de Janeiro 21941-902, Brazil; 6 Laboratory of Clinical Research on Dermatozoonoses in Domestic Animals, Evandro Chagas National Institute of Infectious Diseases, Fiocruz. Av. Brasil, 4365, Manguinhos, Rio de Janeiro 21040-360, Brazil; 7 CEB-Biological Engineering Centre, University of Minho, Campus de Gualtar, 4710-057 Braga, Portugal; 8 LABBELS (Associate Laboratory, Braga/Guimarães), University of Minho, Campus de Gualtar, 4710-057 Braga, Portugal; 9 Platform for Science, Technology and Innovation in Health-PICTIS, Fiocruz. Av. Brasil, 4365, Manguinhos, Rio de Janeiro 21040-360, Brazil

**Keywords:** preservation, *Sporothrix*, phenotype, genotype, proteomic, stability

## Abstract

Sporotrichosis is a subcutaneous mycosis with worldwide distribution and caused by eight pathogenic species of the *Sporothrix* genus. Different ex situ preservation methods are used around the world to maintain the survival, morphophysiological and genetic traits of fungal strains isolated from patients with sporotrichosis for long terms. The main aim of the present study was to evaluate the survival, phenotypic and genotypic stability of *Sporothrix* strains after preservation on PDA slant stored at 4 °C, sterile water and cryopreservation at −80 °C, for a period of time of 6, 12, 18 and 24 months of storage. Eight clinical *Sporothrix* isolates were identified based on a polyphasic approach consisting of classical macro- and micro-morphological traits, biochemical assays, proteomic profiles by MALDI-TOF MS and molecular biology. According to the final identification, one strain was identified as *S. schenckii* (CMRVS 40428) and seven strains were re-identified as *S. brasiliensis* (CMRVS 40421, CMRVS 40423, CMRVS 40424, CMRVS 40425, CMRVS 40426, CMRVS 40427 and CMRVS 40433). In addition, it was observed that the isolates survived after the different time points of storage in distilled water, PDA slant and cryopreservation at −80 °C. For fungi preserved in water, low polymorphisms were detected by the partial sequencing of β-tubulin. Cryopreservation at −80 °C induced morphological changes in one single isolate. The proteomic profiles obtained by MALDI-TOF MS after preservation showed differences among the methods. In conclusion, preservation on agar slant stored at 4 °C was the most effective method to preserve the eight clinical *Sporothrix* strains. This method produced less change in the phenotypic traits and kept the genetic integrity of all strains. Agar slant stored at 4 °C is a simple and inexpensive method and can be especially used in culture collections of limited funding and resources.

## 1. Introduction

*Sporothrix* is a fungal genus distributed worldwide in the tropical and subtropical regions and is composed of about 60 saprophytes species. Among them, until 2006, only *S. schenckii* was considered the only causal agent of the sporotrichosis. From phylogenetic studies, *S. schenckii* was then classified as a species complex, so far composed of *S. schenckii sensu stricto*, *S. brasiliensis*, *S. globosa*, *S. luriei*, *S. mexicana*, *S. pallida*, *S. chilensis* and *S. humicola* [1,2,3,4,5].

Sporotrichosis is a subcutaneous mycosis that has a worldwide distribution and is widely spread in Rio de Janeiro State (Brazil), a region of hyperendemicity with zoonotic transmission by cats. In this region, from 1998 to 2015, more than 5000 human cases of sporotrichosis were recorded, as well as 5113 feline cases from 1998 to 2018 [6,7,8].

Aspects related to the taxonomy, physiology and virulence of *Sporothrix* spp. have been studied over time. Long-term preservation methods to maintain these fungi are required. The best preservation method should guarantee the fungal culture recoverage, purity, phenotypic and genotypic stability, preserving the same traits of the original culture.

The biological material preserved in culture collections must be available to be used in different areas of science, such as for research proposals or for taxonomy in case of epidemiological issues [9]. For choosing a fungal preservation method, both the biological characteristics of the fungal species as well as the resources available in the laboratory must be considered [10].

Continuous subculture preservation is time consuming, prone to contamination, and could suffer alterations due to constant handling. However, when cultures are stored between 4–8 °C, the frequency of phenotypic and genotypic modifications in subcultures is reduced. Consequently, pleomorphic changes are decreased by the technique [11].

Preservation in sterile water at room temperature was originally described by Castellani [12] and it is easy, simple and described by several authors as capable of keeping viable strains without morphological alterations. As an example, *S. schenckii* strains were maintained for more than 15 years without changes in morphological characteristics [13,14,15,16,17,18,19]. In addition, *S. schenckii*, *S. brasiliensis*, *S. mexicana* and *S. globosa* strains kept stability on saline solution stored at 4 °C, for a period of 9 months [20].

In order to keep the strains for a longer time, freeze-drying and cryopreservation are the methods recommended and largely chosen for fungal preservation in many culture collections. Both methods minimize the risks of genetic change, although some fungi cannot resist the process [10]. Brilhante et al. [20] evaluated the cryopreservation method at −80 °C to maintain 20 *Sporothrix* spp. strains over a period of nine months. The authors observed a reduction in conidia counts after fungal storage in this method. In contrast, fungal recovery had no alterations.

Although some studies have demonstrated the integral maintenance of viability and morphological characteristics, most of these studies were carried out before the description of a new pathogenic species of *Sporothrix* spp. In these cases, genotypic and proteomic stabilities were not evaluated after preservation [14,16,20]. In addition, to the best of our knowledge and based on information available in the scientific literature, to date there have been no studies regarding the best morphological phase to preserve *Sporothrix* spp.

The main aim of the present study was to evaluate the survival, phenotypic and genotypic stability of *Sporothrix* strains after preservation on PDA slant stored at 4 °C, sterile water and cryopreservation at −80 °C, for a period of time of 6, 12, 18 and 24 months of storage.

## 2. Materials and Methods

### 2.1. Strains

Eight strains isolated from human patients with sporotrichosis were used in this study: CMRVS 40421 (IPEC 47514), CMRVS 40423 (IPEC 47535), CMRVS 40424 (IPEC 47547), CMRVS 40425 (IPEC 47550), CMRVS 40426 (IPEC 47551), CMRVS 40427 (IPEC 47557), CMRVS 40428 (IPEC 47559) and CMRVS 40433 (IPEC 47553). At the beginning of the present study, all of the *Sporothrix* spp. strains were identified as *Sporothrix schenckii lato sensu*. Fungal identification was performed in the Laboratory of Mycology (Evandro Chagas National Institute of Infectious Diseases/Fiocruz) and was based on classical macro- and micro-morphology. Fungal cultures were then stored at the Collection of Reference Microorganisms in Health Surveillance (National Institute for Quality Control in Health/Fiocruz, Rio de Janeiro, RJ, Brazil). Therefore, from here on to simplify the text, the CRMS numbering will be used.

### 2.2. Preservation Methods

Three methods of fungal preservation were performed and evaluated in the period between 6 and 24 months of storage on (a) potato dextrose agar (PDA, Difco^TM^ Becton, Dickinson and Company, Sparks, NV, USA) slant kept at 4 °C; (b) distilled water (Castellani’s method) kept at room temperature (28 to 30 °C) [12]; and (c) cryopreservation at −80 °C on brain heart infusion broth (BHI, Difco^TM^ Becton, Dickinson and Company, Sparks, NV, USA).

After 6, 12, 18 and 24 months of storage, fungal strains’ recoverage and morphological and biochemical stabilities were assessed, while proteomic and genotypic stabilities were evaluated after 24 months of storage. Before performing preservation, *Sporothrix* isolates were grown on PDA, at 30 °C for 2 weeks in the filamentous form.

Overall, for all preservation methods of filamentous form, plugs of each fungal colony reach in conidia were used, in accordance with the methodology previously described elsewhere [11,12], while for the yeast phase, portions of cultures were obtained with a handle and a concentration of 10^6^ cells was used.

Preservation on PDA slant took place after inoculation of a fragment of each isolate on sterile cryotubes (1.8 mL) containing 1 mL PDA. Slants were incubated at 30 °C for 7 days with a daily evaluation of fungal growth. Slants were then stored at 4 °C [11].

For strain preservation on distilled water, the protocol described by Castellani (1939) with modification was used. Briefly, a small fragment (approximately 5 mm x 10 mm) of each PDA culture medium inoculated with *Sporothrix* sp. was transferred to sterile glass vials containing 4 mL of sterilized distilled water. Vials were sealed with rubber stoppers and stored at room temperature (28 °C to 30 °C) [21]. For cryopreservation of the yeast phase, cells were transferred into cryotubes (1.8 mL) containing BHI supplemented with 30% glycerol and stored at −80 °C [22].

### 2.3. Recoverage

For recoverage assessment, the isolates were removed from storage at the specified periods [20,23]. From the isolates preserved on agar slant stored at 4 °C, a fragment of the culture was subcultured on PDA and incubated at 30 °C for 2 weeks. For recovering strains preserved in distilled water, 30 μL of each suspension were collected and subcultured on PDA and Difco^TM^ Mycosel, and incubated at 30 °C for 2 weeks. Strains cryopreserved at −80 °C were recovered by subculturing 50 μL of original suspension on BHI agar. These isolates were incubated at 37 °C for 7 days. Then, as performed for the other techniques, to convert the isolates into a filamentous phase, the yeast colonies were inoculated on PDA at 30 °C for 2 weeks [22]. For the recoverage analysis, the colony-forming units (CFU) were counted per milliliter.

### 2.4. Phenotype Stability

Phenotypic stability was evaluated according to the *Sporothrix* species identification key proposed by Marimon [1]. For the micromorphological analysis of isolates, conidia were grown on Difco^TM^ cornmeal agar (CMA) and incubated at 30 °C for 12 days.

Colony diameters (in millimeters) were measured from the inoculation of c.a. 1 mm diameter fragment of each culture on Petri dishes containing PDA and incubated at 30 °C for 21 days. Thermotolerance was evaluated by measuring colony diameters at 7 and 21 days after inoculation.

For the carbohydrate assimilation tests, *Sporothrix* isolates were inoculated on Difco^TM^ Yeast Nitrogen Base culture medium (YNB), supplemented with carbohydrate sources such as 0.5% glucose, 0.5% sucrose, and 0.5% raffinose. YNB medium without carbohydrates sources was used as a negative control. Experiments were performed at least three times on different days [1,24].

### 2.5. Genotypic Stability

In order to analyze genotypic stability and the conditions of PCR, partial sequencing of the nuclear β-tubulin gene was assessed as previously described by Oliveira et al. [25,26]. Genomic DNA extraction from the mycelial phase was performed at the time point zero and after 24 months of storage. The chloroform/isoamyl alcohol method as described by Oliveira et al. [24] was used.

For each reaction, the PCR mix consisted of 100 ng DNA, Buffer 10× PCR, 2,0 mM de MgCl_2_ (Invitrogen, Carlsbad, CA, USA), dNTP mix 200 μM (Invitrogen, Carlsbad, CA, USA), 1U de *Taq* Platinum DNA polimerase (Invitrogen), 10 µM of each primer *Bt2-F* (5′GG(CT)AACCA(AG)AT(ATC)GGTGC(CT)GC(CT)3′) and *Bt2*-R (5′ACCCTC(AG)GTGT AGTGACCCTTGGC3′) [27,28]. The amplification program included 35 cycles and an annealing temperature of 64.5 °C.

Sequences from DNA strands were edited with the Codon Code Aligner ver.90.2 software package (Codon Code Corporation, Centerville, MA, USA), followed by alignment by means of the Mega version 4.0 [28]. Sequences were compared by BLAST (Basic Local Alignment Search Tool-NIH) with sequences deposited from NCBI GenBank database (*Sporothrix* AM116914, AM116946, AM498343, AM747289, AM116966, AM498344 and KP711814).

Phylogenetic analyses were performed by the Mega version 4.0 and the phylogenetic relationships among isolates were evaluated using the neighbor-joining method [29]. The significance assessment of the tree was performed using the bootstrap confidence test with 1000 replicates [30]. The construction of the phylogenetic tree was performed in the program BioNumerics 5.0.

### 2.6. Proteomic Stability

Proteomic profiles of the yeast phase isolates were evaluated by MALDI-TOF MS, according to Oliveira et al. [31]. Yeast cells (c.a. 10^6^ cell/sample) were transferred from each culture plate to a 500 µL tube containing 20 µL of 25% formic acid in water (*v*/*v*). Samples were then shaken for 20 s and kept at room (28–30 °C) temperature for 15 min, followed by incubation at 4 °C for 3 h.

The supernatant (1 μL) from each sample was transferred to a paraffin film surface and 2 μL of α-cyano-4-hydroxycinnamic acid matrix (CHCA, Fluka, Buchs, Switzerland) saturated in a solution composed of 33% ethanol, 33% acetonitrile, 31% H_2_O and 3% trifluoroacetic acid (TFA) was added and mixed gently. Then, four samples of each isolate (1 μL) was spotted in triplicate onto a stainless MALDI-TOF MS plate (FlexiMass™, Shimadzu Biotech, Manchester, UK).

Proteomic evaluation was performed using SARAMIS^®^ software (Spectral Archiving and Microbial Identification System, AnagnosTec, Postdam, Germany).

### 2.7. Statistical Analysis

Analysis of the conidial morphology and carbohydrate assimilation profile was performed by description and comparison of absolute counts. The average of the colony diameters (at 30 °C and 37 °C) was assessed for colonies of all strains at 0, 6, 12, 18 and 24 months by using the Friedman non-parametric test. In addition, to identify differences in each group in relation to the standard group (0 months), the Wilcoxon test with Bonferroni correction was used. Statistical significance was set at *p* < 0.05 (software R Core Team 2016).

## 3. Results

### 3.1. Phenotypic Characterization before Preservation

After 21 days of incubation at 30 °C on PDA, *Sporothrix* sp. isolates showed colonies in shades of light or dark brown, with a velvety or glabrous texture. Microscopic examination revealed hyaline and septate hyphae, intermediate or terminal conidia in sympodial conidiophores along the hyphae, with variable morphology (obovoid or piriform, globose and triangular). Only the strain *S. schenckii* CMRVS 40428 developed triangular and pigmented conidia.

At the same observation point, the diameter of colonies on PDA ranged from 22 to 63 mm, the latter being the measure of the colony of the isolate CMRVS 40428. At 37 °C, colony growth was lower, with diameters ranging from 6.50 to 11.50 mm. The median diameter of colonies preserved after 21 days of incubation at 30 °C and 37 °C did not show a significant difference (*p* < 0.05) (Table 1).

**Figure 1:**
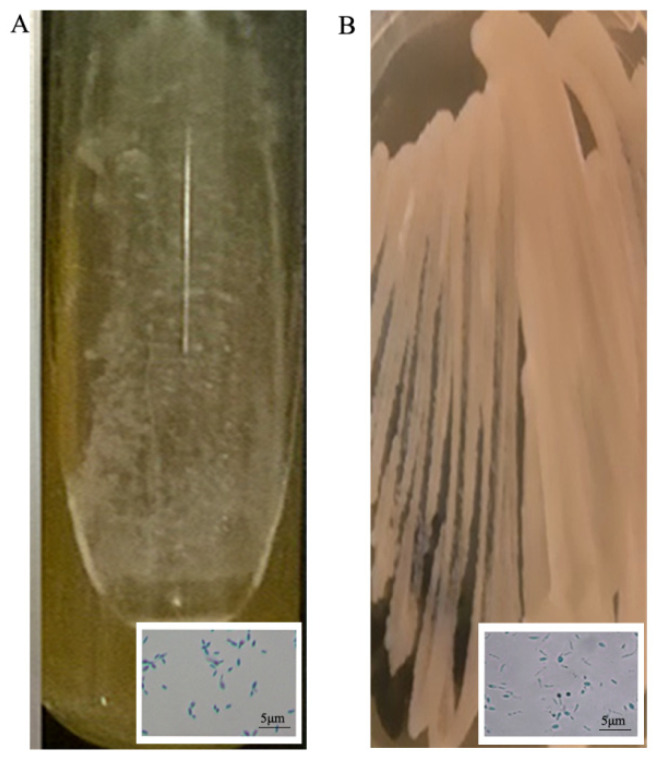
Morphological differences between the *Sporothrix* strain CMRVS 40433 in yeast phase before (**A**) and after (**B**) cryopreservation at −80 °C for 24 months.

All preserved isolates remained able to grow after storage on all of the methods and times assessed. A mean recovery of 1.47 × 10^5^ and 2.38 × 10^5^ CFU/mL was obtained for *S. brasiliensis* and *S. schenckii* strains used in this study, respectively. The macro- and micro-morphological characteristics of all fungal strains were consistent after preservation on an agar slant at 4 °C and sterile water. After cryopreservation storage at −80 °C, macroscopic and microscopic analysis revealed alteration in some isolates. After cryopreservation at −80 °C, analysis of the macro- and micro-morphology of some isolates in the filamentous phase revealed alterations in their ability to dimorphism. Regarding the isolates in the yeast phase, alterations were observed only in the microscopic analysis (Figure 1A,B).

**Figure 2:**
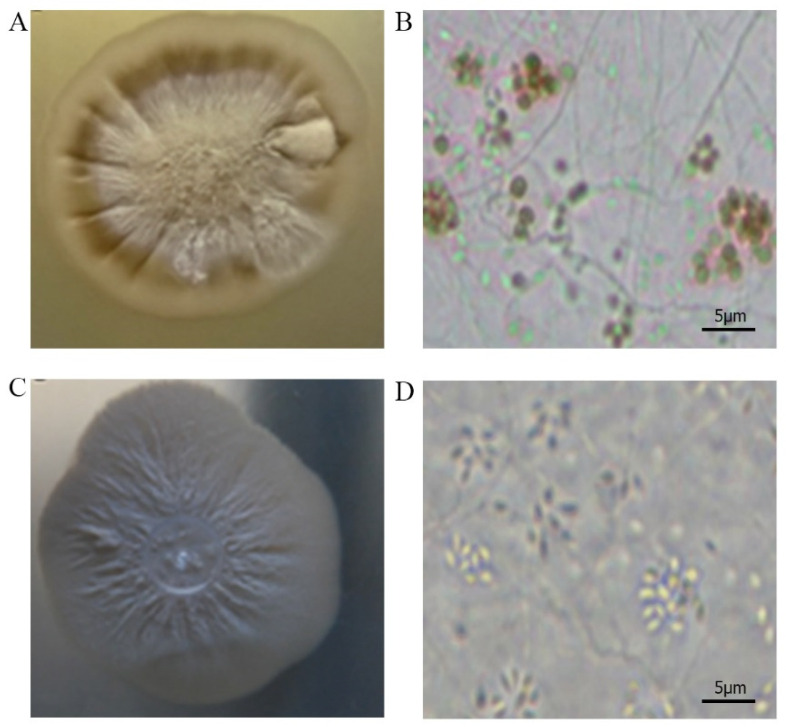
Morphological differences of the *Sporothrix* strain CMRVS 40433 before and after cryopreservation at −80 °C for 24 months. (**A**) Pigmented colony and (**B**) globose pigmented conidia before preservation; (**C**) non-pigmented colony and (**D**) hyaline conidia after preservation.

*Sporothrix brasiliensis* strain CMRVS 40433, for instance, which previous to preservation presented a brown colony and pigmented conidia (Figure 2A,B), after storage showed a white colony and hyaline conidia (Figure 2C,D).

The biochemical profile showed that all *Sporothrix* isolates were able to assimilate glucose and remain stable after storage. Sucrose was the carbohydrate that presented the greatest variation: three *S. brasiliensis* isolates (CMRVS 40421, 40423 and 40425) could be identified according to the previously described identification since they are capable to grow in the presence of this carbon source after storage in all preservation methods. Only the *S. schenckii* isolate CRMVS 40428 started to assimilate raffinose after 18 and 24 months of cryopreservation at −80 °C (Table 2).

### 3.2. Genotypic Stability

Genotypic stability was analyzed by partial sequencing of the β-tubulin gene. A high agreement degree was observed between preservation in distilled water at 4 °C and cryopreservation at −80 °C. After storage, all *S. brasiliensis* strains were grouped into the same cluster with 98% similarity. The MP tree was obtained using the close neighbor interchange algorithm with search level 2 in which the initial trees were obtained with the random addition of sequences (10 replicates). The tree is drawn to scale, with branch lengths calculated using the average pathway method, and are in the units of the number of changes over the whole sequence. The codon positions included were 1st + 2nd + 3rd + noncoding. All positions containing gaps and missing data were eliminated from the dataset (complete deletion option) (Figure 2).

**Figure 3:**
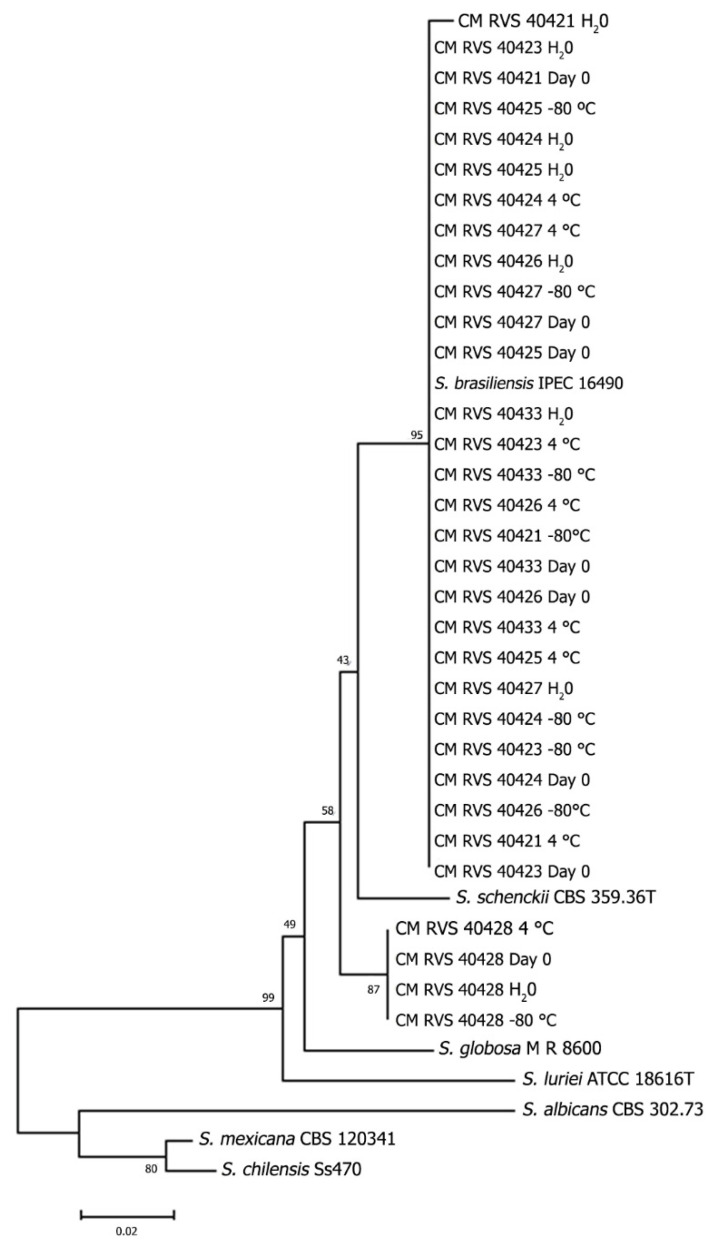
Genotypic stability. Evolutionary relationships of 36 taxa. The evolutionary history was inferred using the Maximum Parsimony (MP) method. Phylogenetic analyses were conducted in MEGA4. The phylogenic tree of partial β-tubulin gene performed for the eight isolates at day zero and after 24 months of preservation. H_2_O = preservation in distilled water; 4C = preservation at 4 °C; −80 = cryopreservation at −80 °C.

The *Sporothrix schenckii* strain CMRVS 40428 was also grouped in the same cluster regardless of the three methods used (Figure 3). However, the *S. brasiliensis* strains CMRVS 40421 and CMRVS 40433 showed base pair substitution after preservation in distilled water. After storage, the *S. brasiliensis* strain CMRVS 40421 showed a polymorphism of 2 bp (positions 34 G/C and 92 G/A); while the *S. brasiliensis* strain CMRVS 40433 showed a polymorphism of 1 bp (positions 66 A/T). In the other preservation methods, agar slant stored at 4 °C and cryopreservation at −80 °C, there were no changes in the DNA sequences of the β-tubulin gene for any assessed strains.

### 3.3. Proteomic Stability

**Figure 4:**
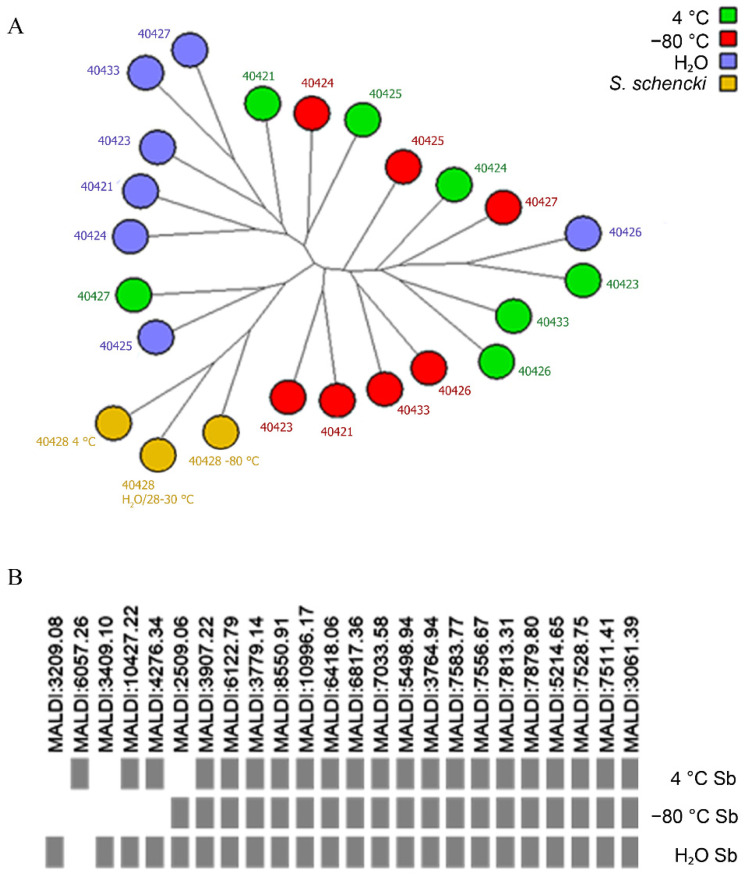
(**A**) Phylogenetic tree neighbor joining based on Pearson correlation constructed with spectra obtained by MALDI-TOF MS of 8 isolates distributed according to species and each of the three preservation methods; (**B**) proteomic profile of MALDI-TOF MS for an isolate of *S. brasiliensis* CMRVS 40424 (IPEC 47547) selected to exemplify the differences in the proteomic profile of the same isolate preserved by different methods. The rectangles in gray color indicate the presence of protein spectra, with molecular weight indicated on the side.

The seven strains: CMRVS 40421, CMRVS 40423, CMRVS 40424, CMRVS 40425, CMRVS 40426, CMRVS 40427 and CMRVS 40433 were identified by MALDI-TOF MS as *S. brasiliensis* and the strain CMRVS 40428 was identified as *S. schenckii* (Figure 4A). For fungal identification, MALDI-TOF mass spectra were compared with spectra archived in an in-house database using the SARAMIS^®^ software. The in-house database was produced and fed with MALDI-TOF spectra of the reference and well-identified strains, by molecular biology. In the SARAMIS^®^ software, fungal identification is based on a confidence percentage, where the fungal identification reliability ranges from 60 to 99.99%.

In the present work, before and after preservation, for all fungal isolates MALDI-TOF MS identification presented confidence percentage varying from 70 to 99.99%. When compared to genotypic identification, some differences in the distribution of isolates by clade were observed (Figure 4). Nevertheless, the preservation regime did not affect the confidence percentage for any fungal isolate. MALDI-TOF spectra variability was observed for the strain *S. brasiliensis* CMRVS 40424. In this case, few molecular masses in the proteomic analysis were absent depending on the preservation method (Figure 4B). However, this did not affect the confidence percentage and fungal identification. No variability was observed in the genotypic analysis for this strain *S. brasiliensis* CMRVS 40424 (Figure 3).

## 4. Discussion

Sporotrichosis is a subcutaneous mycosis of worldwide distribution, caused by pathogenic species of the *Sporothrix* complex [1,6]. Sporotrichosis is commonly described based on the use of polyphasic taxonomy, which brings together methodologies of classical taxonomy combined with the use of molecular tools.

The description of new *Sporothrix* species as causal agents of sporotrichosis reinforces the need for accurate identification of species and their maintenance in public service fungal collections for future studies. However, there are just a few studies on the preservation of *Sporothrix* spp. In addition, studies available in the literature do not use molecular and proteomic tools to evaluate the fungal strains’ characteristics after their long-term preservation on different methods.

In the present study, few *Sporothrix* strains could be identified based on the identification key proposed by Marimon et al. [1]. The *S. schenckii* CMRVS 40428 strain showed colony diameters greater than 50 mm (63 mm) at 30 °C, a phenotypic characteristic of *S. mexicana*, *S. pallida* or *S. luriei* [1]. Based on the molecular analysis, the *S. schenckii* CMRVS 40428 strain grouped together into the *S. schenckii* clade. Similar data were also observed and described by Oliveira et al. [24], who reported greater colony diameters for *S. schenckii* strains grown at 30 °C, reinforcing the need to use polyphasic taxonomy to identify *Sporothrix* species.

The carbohydrate assimilation profile was the methodology whose results showed greater disagreement. Only three *Sporothrix* strains (CMRVS 40421, 40423 and 40425) were correctly identified according to the carbohydrate assimilation profiles. Other authors have also reported that identification based only on phenotypic traits may be inconclusive or present ambiguous results [24,32,33]. It suggests that identification discrepancies are associated with phenotypic variability within species. Herein, it is the first time that the impact of three different long-term storage methods on the recoverage, phenotype, and genotypic stabilities of *Sporothrix* spp. strains are analyzed.

All preservation methods were able to keep the isolates recoverable. Preservation in sterile water (Castellani’s method), was found to be the simplest and most cost-effective method for long-term storage of *Sporothrix* spp., keeping the survival, phenotype and genotypic stabilities of the assessed fungal cultures [15,18,19].

In 1992, Borba et al. [13] published a study in which four strains of *S. schenckii* were kept in distilled water for 23 years and were recovered on Sabouraud glucose agar medium. According to the authors, fungal strains remained able to grow with unaltered morphological characteristics [13].

Brilhante et al. [20] evaluated the maintenance of different *Sporothrix* spp. strains stored on agar slant at 4 °C, frozen at −20 °C, and cryopreserved at −80 °C for 3, 6 and 9 months. No changes were observed in the fungal macro- and micro-morphological characters. However, other relevant characteristics such as carbohydrate assimilation and thermotolerance were not analyzed [20].

In our study, *Sporothrix* spp. strains cryopreserved at −80 °C showed changes after storage. Loss of both colony color and conidia pigmentation was observed for the strain *S. brasiliensis* CMRVS 40433. This finding is in accordance with data obtained by Marimon et al. [1]. According to these authors, the type strain *S. schenckii* CBS 359.36, which traditionally produces pigmented conidia, only formed sympodial and hyaline conidia after long-term storage. It is necessary to emphasize that, in the present study, the morphological changes occurred in the yeast phase, and the characteristics of the fungus preserved in its filamentous phase were kept.

Borman et al. [17] also observed alterations in up to 20% of isolates of dermatophyte species after preservation in water and showed that the main alteration was the reduction of conidiogenesis, which produced sterile mycelium and non-pigmented colonies. These modifications prevented the identification of isolates based on macro-morphology after storage. In contrast, in the present study, the changes were not sufficiently severe to compromise the identification of the fungus *S. brasiliensis* CMRVS 40433 based on its macro- and micro-morphological characteristics.

The average diameter of fungal colonies grown at 30 °C and 37 °C did not change after storage, as well as thermotolerance was preserved at both storage temperatures. The results obtained in the present study are in agreement with data available in the literature [16], where five *Sporothrix* strains preserved in sterile water and continuous subculture for 18 years were further cultivated at 35 °C and 37 °C and were able to complete the dimorphism.

Lima and Borba [23] evaluated the stability of dimorphism in strains of *Sporothrix schenckii lato sensu* preserved under mineral oil and in soil over a period of 8 to 49 years. *Sporothrix schenckii lato sensu* strains were able to complete the entire dimorphic process. This fungal species showed a great survival rate after long-term storage under mineral oil.

Barreira et al. [34], performed morphophysiological and molecular studies of *Sporothrix* strains preserved under mineral oil over a period of 34 to 64 years. According to the results obtained, one *Sporothrix* strain lost the ability to sporulate, despite it can be reverted by using a medium culture supplemented with rose bush branches. These data demonstrate that the choice and evaluation of storage methods are important for the successful preservation of *Sporothrix* species [23].

In the present study, data regarding carbohydrate assimilation presented alterations that diverge from the taxonomic identification key of the species proposed by Marimon et al. [1]. Herein, three strains that grouped in the *S. brasiliensis* clade (CMRVS 40421, CMRVS 40423 and CMRVS 40425) started to assimilate sucrose after storage on all preservation methods assessed.

In addition, the strain *S. schenckii* CMRVS 40428, which did not assimilate raffinose before cryopreservation at −80 °C, after 18 and 24 months of storage, started to assimilate this carbohydrate. Mendoza et al. [16] also reported changes in the ability to hydrolyze starch by *Sporothrix schenckii lato sensu* strains after 18 years time storage in distilled water. These results demonstrate that phenotypic alterations can cause errors in the identification of microorganisms [35,36].

Although phenotypic changes were observed after storage, the genotypic stability assessed by partial sequencing of the β-tubulin gene showed that storage methods preserved genetic integrity. Few changes were observed in the *Sporothrix* strains CMRVS 40421 and CMRVS 40433 after preservation in distilled water. These corroborate the results of Broughton et al. [37], who also observed genetic polymorphisms, evaluated by PCR fingerprints, after cryopreservation in liquid nitrogen.

It is relevant to evaluate whether the alteration in the β-tubulin gene can promote alterations in the isolates, since this gene constitutes a protein that structures microtubules and it is the main component of the cytoskeleton of eukaryotic cells involved in many essential processes, including cell division and intracellular transport [38,39,40,41].

In addition, they are important in morphogenesis in dimorphic fungi and in the growth of hyphae of various pathogenic fungi. Zhang et al. [42] highlighted the importance of β-tubulin as part of the microtubule since the decrease in growth and virulence of the fungus *Metarhizium acridum* can be explained in part by an induced mutation in the β-tubulin gene.

Previous works have reported a decrease in the virulence of fungal strains proportional to an increase in preservation time, which can be restored after passage in an animal model [43,44,45]. Nevertheless, these studies were not related to *Sporothrix* species. Based on the results obtained in the present study, strains did not lose the dimorphic capacity, which could be an indicator of keeping their virulence. However, additional studies are required to understand the impact of preservation on fungal virulence.

Regarding proteomic stability, after 24 months, all isolates persevered on agar slant at 4 °C, on distilled water, and under cryopreservation at −80 °C, were identified by MALDI TOF MS, corroborating the results obtained with the partial sequencing of the β-tubulin. Proteomic profiles by MALDI-TOF MS were used by Lima-Neto et al. [46] to identify isolates of *Candida* spp. preserved for a period from 1 to 52 years in a service culture collection. According to the authors, 15% of assessed strains showed discordance when compared with morphological and biochemical analyses, but the sequencing of the ITS region confirmed the identification obtained by MALDI-TOF MS, demonstrating that this mass spectrometry technique is a reliable tool to identify fungal species preserved in service culture collections. In the present study, the evaluation of the stability of the proteomic profiles by MALDI-TOF MS showed a difference regarding all preservation methods assessed.

Rodriguez et al. [47] assessed the effect of freeze-drying and long-term storage on the biotechnological potential of 12 strains of *Aspergillus* section *Nigri*. Twelve fungal strains were analyzed before and after being freeze-dried and aged by accelerated storage, at 37 °C in the dark for 2 and 4 weeks. According to the authors, the proteomic fingerprint by MALDI TOF MS showed that strains and species were quite stable after preservation. Once the proteomic profiles by MALDI-TOF MS are mainly composed of fungal ribosomal proteins, this analytical technique gives good indications of the impact that preservation and long-term storage might have on these target proteins [47].

## 5. Conclusions

The present study demonstrates for the first time the impact of three different preservation methods on the phenotypic and genotypic characters of *S. schenckii* and *S. brasiliensis* stored for up to two years in culture collections. In fact, as a pioneering study assessing the impact of three preservation methodologies simultaneously, it reflects the scarcity of comparative data from other authors. The long period for the evaluation of alteration (24 months) makes it difficult to insert new experiments/analyses and constitutes a limitation of this study. Despite this, the results presented herein demonstrate that the strains *S. schenckii* CMRVS 40428 and *S. brasiliensis* CMRVS 40421, CMRVS 40423, CMRVS 40424, CMRVS 40425, CMRVS 40426, CMRVS 40427 and CMRVS 40433 were viable after preservation on agar slant at 4 °C, distilled water and cryopreservation at −80 °C for 24 months of storage.

Phenotypic changes were observed in both distilled water and cryopreservation methods, compromising the identification of isolates. However, the phenotypic characteristics and genetic integrity of *S. schenckii* and *S. brasiliensis* strains were kept by preservation on an agar slant at 4 °C. It indicates that this method was more effective to preserve these fungal species.

Agar slant at 4 °C can be suggested as an alternative method for the preservation of *Sporothrix* spp., in culture collections with limited resources, as it is a simple and cost-effective method. However, for a wide variety of *Sporothrix* species preserved in culture collections, their evaluation as a standard methodology is necessary.

## Figures and Tables

**Table 1:** Average diameter of *Sporothrix* strains colonies grown at 30 and 37 °C after storage in the three different preservation methods.

				Diameter of Colonies (mm)			*p*-Value
		Preservation Method	Time of Preservation (Months)	Min	Median	Max	
Growth temperature	30 °C	H_2_O	6	19.00	41.25	50.00	1.000
			12	21.00	31.00	49.50	0.800
			18	21.00	36.25	46.25	1.000
			24	18.25	39.38	54.00	1.000
		4 °C	6	22.00	36.50	63.00	**
			12	20.00	32.50	40.50	0.310
			18	17.25	33.50	45.75	0.940
			24	19.75	37.50	53.75	1.000
		−80 °C	6	21.25	29.50	49.00	0.140
			12	25.00	34.00	42.50	1.000
			18	21.25	41.12	46.75	1.000
			24	28.25	41.62	51.25	1.000
	37 °C	H_2_O	6	8.00	11.00	14.50	0.740
			12	6.00	9.25	12.00	1.000
			18	7.25	10.12	12.50	1.000
			24	6.25	8.79	11.00	0.800
		4 °C	6	6.50	10.50	11.50	**
			12	6.00	7.00	11.50	1.000
			18	6.75	8.38	12.75	1.000
			24	5.25	9.25	72.75	1.000
		−80 °C	6	4.50	9.62	15.50	1.000
			12	4.50	8.50	10.50	1.000
			18	7.25	9.62	10.50	1.000
			24	8.25	11.30	12.75	1.000

Correlation of colony diameter before the storage at 30 °C and 37 °C. H_2_O = preservation in distilled water; 4 °C = preservation at 4 °C; −80 °C = cryopreservation at −80 °C. ** The colony diameters were constant after six months of storage at 4 °C.

**Table 2:** Alterations in sucrose assimilation after storage.

		H_2_O				4 °C				−80 °C			
Isolate	T0	6M	12M	18M	24M	6M	12M	18M	24M	6M	12M	18M	24M
CMRVS 40421	-	+	+	+	+	-	+	+	+	+	+	+	+
CMRVS 40423	-	+	+	+	+	-	-	+	+	-	+	+	+
CMRVS 40425	-	-	-	+	+	-	-	+	+	-	-	+	+

Carbohydrate assimilation test (+ for positive and – for non-assimilation) was performed at the time of isolation (T0) and after 6, 12, 18 and 24 months of preservation (6M, 12M, 18M and 24M). H_2_O = distilled water preservation method; 4 °C = refrigerated preservation method at 4 °C; −80 °C = cryopreservation method at −80 °C.

## Data Availability

Not applicable.
